# Exploring laser-induced acute and chronic retinal vein occlusion mouse models: Development, temporal in vivo imaging, and application perspectives

**DOI:** 10.1371/journal.pone.0305741

**Published:** 2024-06-17

**Authors:** Xiaowei Xu, Xun Li, Qingqing Tang, Yi Zhang, Li Zhang, Meixia Zhang

**Affiliations:** Department of Ophthalmology and Research Laboratory of Macular Disease, West China Hospital, Sichuan University, Chengdu, Sichuan, China; Shinshu University School of Medicine, JAPAN

## Abstract

Photodynamic venous occlusion is a commonly accepted method for establishing mouse models of retinal vein occlusion (RVO). However, existing model parameters do not distinguish between acute and chronic RVO subtypes. Large variations in laser energy seem to correlate with fluctuating retinopathy severity and high rates of venous recanalization during the acute phase, along with the variable levels of retinal perfusion during the chronic phase. After optimizing the modeling procedure and defining success and exclusion criteria, laser energy groups of 80mW, 100mW, and 120mW were established. Multimodal imaging confirmed that higher energy levels increased the incidence of retinal cystoid edema and intraretinal hemorrhage, exacerbated the severity of exudative retinal detachment, and reduced the venous recanalization rate. For the acute model, 100mW was considered an appropriate parameter for balancing moderate retinopathy and venous recanalization. Continuous imaging follow-up revealed that day 1 after RVO was the optimal observation point for peaking of retinal thickness and intensive occurrence of retinal cystic edema and intraretinal hemorrhage. After excluding the influence of venous recanalization on retinal thickness, acute retinal edema demonstrated a positive response to standard anti-vascular endothelial growth factor therapy, validating the clinical relevance of the acute RVO model for further study in pathogenic mechanisms and therapeutic efficacy. For the chronic model, the 120mW parameter with the lowest venous recanalization rate was applied, accompanied by an increase in both photocoagulation shots and range to ensure sustained vein occlusion. Imaging follow-up clarified non-ischemic retinopathy characterized by tortuosity and dilation of the distal end, branches, and adjacent veins of the occluded vein. These morphological changes are quantifiable and could be combined with electrophysiological functional assessment for treatment effectiveness evaluation. Moreover, the stable state of venous occlusion may facilitate investigations into response and compensation mechanisms under conditions of chronic retinal hypoperfusion.

## Introduction

RVO is a leading cause of vision impairment in the middle-aged and elderly population, second only to diabetic retinopathy [1]. Photodynamic occlusion, achieved through intravenous injection of a photosensitizer combined with laser photocoagulation, stands as a widely accepted method for RVO induction in murine models [2]. The process involves activating the photosensitizer at its peak absorption wavelength with the laser to generate oxygen singlets, resulting in damage to the venous endothelium and initiating platelet adhesion and aggregation for photothrombosis [3]. Rose Bengal is a frequently selected photosensitizer, with doses centered around 40mg/kg. However, the laser energy required to occlude a vein varies considerably in reported murine RVO models, ranging from 100mJ to over 3000mJ, influenced by three key laser parameters: power, duration, and number of shots (S1 Table).

RVO encompasses acute and chronic subtypes. Acute RVO is characterized by tortuous dilated veins, intraretinal hemorrhage, retinal edema, and cotton-wool spots, while chronic RVO primarily exhibits vasculature changes of retinal nonperfusion, neovascularization, and associated complications [4]. The existing RVO model parameters do not differentiate between acute and chronic subtypes; instead, the distinction relies on the duration of the observation window for a given parameter. The acute phase lasts for about 1 week, marked by retinal edema and hemorrhage with various severity. Clinical-relevant intraretinal hemorrhage may be substituted with subretinal or diffuse hemorrhage [5–8], or a more common underreaction of absence of hemorrhage. Other prevalent overreactions include severe exudate and excessive ischemia, leading to the detachment of the internal limiting membrane [9] and arteriovenous occlusion [3, 5, 10], respectively. The chronic phase, typically defined as 1 week to 1 month after photocoagulation, exhibits heterogeneity in retinal perfusion levels. Studies diverge on the formation and development of retinal nonperfusion and neovascularization, while this animal model is initially designed to replicate ischemic retinopathy [3, 7, 11–14].

Venous recanalization is a prominent limitation of the RVO murine model, exerting a greater impact during the chronic phase than the acute phase. Venous recanalization initiates at 1 to 3 days after RVO, progressing to 2/3 to all individuals approximately a week later [3, 5–7, 9, 10, 15]. Some studies specified a 24-hour duration of venous occlusion, with subsequent venous recanalization no longer being considered an exclusion criterion [6, 15–18].

We speculate that laser energy influenced RVO retinopathy and suggest adopting different modeling parameters for acute and chronic RVO. Acute RVO targets an appropriate degree of retinopathy, while chronic RVO emphasizes persistent occlusion of the target vein. We aim to optimize the acute RVO mouse model by identifying optimal follow-up time points, refining success and exclusion criteria, defining venous recanalization and assessing its impact. We will also validate the responsiveness of the acute RVO model to classical anti-vascular endothelial growth factor (VEGF) therapy, clarifying its clinical relevance for further research. Furthermore, we will develop the chronic RVO mouse model with a novel photocoagulation method to prolong the duration of venous occlusion and explore changes in retinal vasculature and perfusion. Its potential applications will be explored through a literature review alongside the pathological alterations.

## Materials and methods

### Animals and anesthesia

Male *C57BL/6J* mice, aged 6–8 weeks and weighing 19-21g, were purchased from Dossy Experimental Animals Co.,Ltd., Chengdu, China. The mice were adaptively raised in an environment with a 12h/12h light/dark cycle, constant room temperature of 26°C, and adequate water and food for one week before the experiment. All experimental procedures followed the Statement for the Use of Animals in Ophthalmic and Vision Research of the Association for Research in Vision and Ophthalmology (ARVO) and were under the supervision of the Institutional Animal Care and Use Committee (IACUC) of Sichuan University (Protocol Number: 20220907001). Before RVO modeling and imaging examination, mice were routinely anesthetized by intraperitoneal injection of 1% sodium pentobarbital, 0.15mL/20g, and pupils were dilated by Mydrin^®^ eyedrops (Santen Pharmaceutical Co.,Ltd., Osaka, Japan). The mice were euthanized by cervical dislocation at the end of the intervention.

### Laser photocoagulation for the RVO mouse model

The RVO mouse model was generated using a slit lamp system comprising a slit lamp microscope (66 Vision Tech Co.,Ltd., Suzhou, China) and a Nd:GdVO4 532nm laser photocoagulator (MEDA Co.,Ltd., Tianjin, China). Instrument pre-adjustments included maximizing slit width, setting a 0° light source angle, adjusting microscope magnification to 40X with the focal point aligned with the laser photocoagulator, and placing the laser indicator at the center of the slit lamp illumination. After pupil dilation, slit lamp microscopy was conducted to exclude individuals with refractive media opacity or fundus deformity. Mice were then anesthetized immediately following a tail vein injection of the photosensitizer Rose Bengal (40mg/kg, 8mg/mL). A cover glass coated with viscoelastic agents was applied to flatten the cornea and ensure visibility of the fundus structure. 15 minutes was ensured for photosensitizer systematic circulation when a state of surgical anesthesia was achieved before the laser procedure.

Acute RVO mouse model: The laser parameters settings were powers of 80mW, 100mW, and 120mW, duration of 1s, and spot size of 50μm. 4 laser shots were performed continuously at the same location 2 to 3 papillary diameters (PD) away from the optic disc for one major vein, and a total of 3 veins for each eye. When only one or no vein was occluded, an additional vein was photocoagulated with a total number of no more than four.

Chronic RVO mouse model: The laser parameters settings were power of 120mW, duration of 1s, and spot size of 50μm. Consecutive 10 to 15 laser shots for a target vein started from 3PD from the optic disc towards the proximal end with an occlusion length of 1.5 to 2PD.

Sham laser group: As a control for the acute RVO mouse model, no photosensitizer, identical laser parameters, and 4 continuous laser shots were performed 2 to 3PD from the optic disc avoiding large retinal vessels at each of three sites per eye.

Double-eye modeling was conducted on approximately 10 experimental animals to ensure that each group had 20 to 30 veins successfully occluded after photocoagulation.

### Scanning laser ophthalmoscopy (SLO)

SLO was performed using Heidelberg Spectralis HRA+OCT Fundus Diagnostic Instrument (Heidelberg Engineering, Inc.) with a widefield lens for an en-face view of the normal and RVO retinal morphology. The Multicolor (Mcolor) and Infrared (IR) examination modes (55°×55° field; high-resolution scan mode; automatic real-time mean (ART) up to 100 frames) were applied for the acute model and sham laser group immediately after photocoagulation (day 0) and on the day of 1, 2, 3, 5 and 7 after RVO. The 80mW, 100mW, and 120mW groups were followed up with the same method immediately and on days 1 and 7 after RVO. During the evaluation of the anti-VEGF treatment efficacy in the acute model, IR was performed on days 1 and 2 after RVO. The chronic model only required an IR test immediately and on days 7, 14, 21, and 31 after RVO.

### Fundus fluorescein angiography (FFA)

FFA was implemented through Heidelberg Spectralis HRA+OCT with a widefield lens in fluorescein angiography (FA) mode (55°×55° field; high-resolution scan mode; ART up to 100 frames). Mice were injected intraperitoneally with 0.2 mL of 2% sodium fluorescein after pupil dilation and anesthesia. Venous obstruction was selectively examined 2 min after the injection in the acute model groups, at the time points when the results of SLO were ambiguous. In the chronic model group, venous occlusion and retinal perfusion at days 7, 14, 21, and 31 after RVO were evaluated with no strict requirement for imaging time after contrast agent injection, as the animal posture was needed to constantly adjust for the peripheral retina entering the field of view. Normal posterior pole and peripheral fundus FA imaging were also recorded.

### Optical coherence tomography (OCT)

OCT images of the retinal transverse view were promptly captured for the acute RVO model and the sham laser group post-photocoagulation and at 1, 2, 3, 5, and 7 days after RVO. Normal eyes were also imaged for reference. Additionally, OCT images for the 80mW, 100mW, and 120mW groups were acquired immediately after photocoagulation, as well as at 1 and 7 days after RVO. In accessing the responsiveness to anti-VEGF medications in the acute model, OCT scans were performed post-photocoagulation, and on days 1 and 2 after RVO. After the mice were anesthetized with the pupil sufficiently dilated, Heidelberg Spectralis HRA+OCT entered the IR+OCT combined imaging mode with a standard lens (30°×30° field; high-speed scan mode; ART up to 100 frames). OCT images were collected at the distal tangent of the laser spot after RVO and 3PD off the optic disc in normal eyes as the baseline guided by IR. The option of follow-up was checked during the observation window to ensure that the examination was performed at the same site. OCT images were quantitatively analyzed using Heidelberg Eye Explorer software for retinal layer thickness assessment, with manual correction for retinal delamination results. Follow-up time points with refractive media opacity or severe retinal detachment (RD) were excluded.

### Histology and immunofluorescence

Histology and immunofluorescence were performed on normal eyes of mice to clarify the retinal layers in the longitudinal section and the distribution of retinal capillaries, respectively.

Histology: The mice were euthanized, and the eyeballs were enucleated and fixed in 4% paraformaldehyde. After routine dehydration, the eyeballs were embedded in paraffin, cut into 5 μm slices, and stained with hematoxylin-eosin for observation.

Immunofluorescence: Eyeballs were enucleated and fixed as before, embedded in optimal cutting temperature compound, frozen, and cut into 10μm slices at -20°C. After incubation with isolectin B4 (1:200, Sigma-Aldrich, St Louis, MO, United States) at 4°C overnight and stained with 4’,6-diamidino-2-phenylindole (DAPI), the slices were observed under the fluorescence microscope (Carl Zeiss, Germany).

### Drug administration

Immediately after acute RVO model induction, experimental animals received drug administration via intravitreal injection. A 30-gauge needle punctured 1mm posterior to the nasal limbus, followed by a 34-gauge microsyringe (Hamilton (shanghai) Laboratory Equipment Co.,Ltd.) inserted vertically and advanced approximately 60° towards the optic nerve in the vitreous cavity for about 1mm, after which the drug was slowly and steadily injected. 14 eyes in the experimental group received Aflibercept (Eylea^®^, Bayer AG) at 10 μg/μL, 2μL per eye; 12 eyes in the control group were given an equal amount of phosphate-buffered saline (PBS).

### Statistical analysis

Data were presented as mean±SEM, rate, and percentage. The Chi-square test (followed by Bonferroni multiple comparisons) was employed to compare the rates and percentages among two (or more) groups. The comparison between two groups of samples conforming to normality was conducted by Student’s t-test, with Welch correction applied in case of inequality of variance. The Mann-Whitney U test was employed for datasets not adhering to normality. A P-value of less than 0.05 was deemed to signify statistical significance. The statistical analysis was completed with the software SPSS 25.0 (SPSS, Inc. Chicago, IL, United States).

## Results

### Target vein identification and focus adjustment during the laser procedure

**Fig 1:**
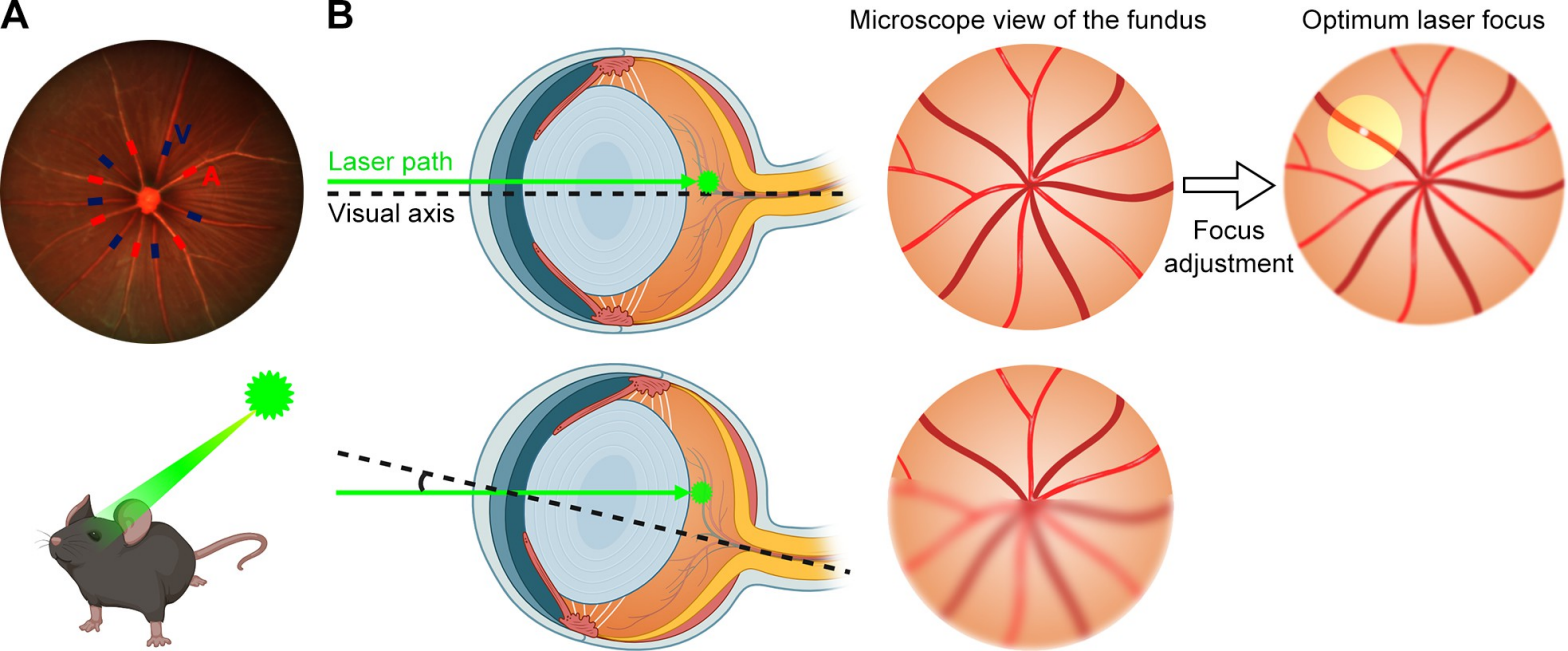
Major vein identification and laser focus adjustment for photocoagulation. (A) Distinction of the major retina arteries and veins at the posterior pole of the fundus. Red and blue marks represented retinal major arteries and veins, respectively. Five to six arteries and veins respectively emanated from the optic disc and were arranged in an alternating spoke-like pattern. Retinal arteries were thin and bright red, with obvious reflective walls, and might send out two to three branches at the posterior pole of the fundus. Retinal veins were thick and dark red, and did not give off branches at the posterior pole of the fundus. (B) Focus adjustment procedure during the photocoagulation. The visual axis was aligned with the laser path for centering the optic disc and clear visibility of major blood vessels. Partial fundus structure blurring caused by misalignment should be avoided. The laser focus was adjusted to target a small segment of the vein at the laser indication point until the wall reflection appeared, while simultaneously blurring the surrounding retinal structure to achieve an optimal photocoagulation condition.

Accurate distinction of major veins from arteries was essential for identifying target ones. SLO image showed 5 to 6 retinal arteries and veins in the mouse retina respectively, each emanating from the optic disc and alternately arranged in a spoke shape. A major retinal artery often had 2 to 3 branches at the posterior pole with a narrower diameter, bright red color, and a more conspicuous wall reflection. Major retinal veins were in dark red with wider diameters and no branches at the posterior pole (Fig 1A). Adjustment of the laser focus constituted a crucial element for achieving successful modeling. The visual axis should be aligned with the light (laser) path by adjusting the contact angle between the cover glass and the cornea, as well as the animal’s posture, ensuring that the optic disc remained at the center of the microscope field with clear visibility of major blood vessels. Partially blurred fundus structure due to misalignment of the visual axis and laser path should be avoided. Subsequently, the laser focus was precisely tuned to achieve an optimal photocoagulation condition: a small segment of the target vein was reflected at the laser indication point, while the remaining retinal structure was slightly blurred (Fig 1B).

### Successful construction of an RVO mouse model

**Fig 2:**
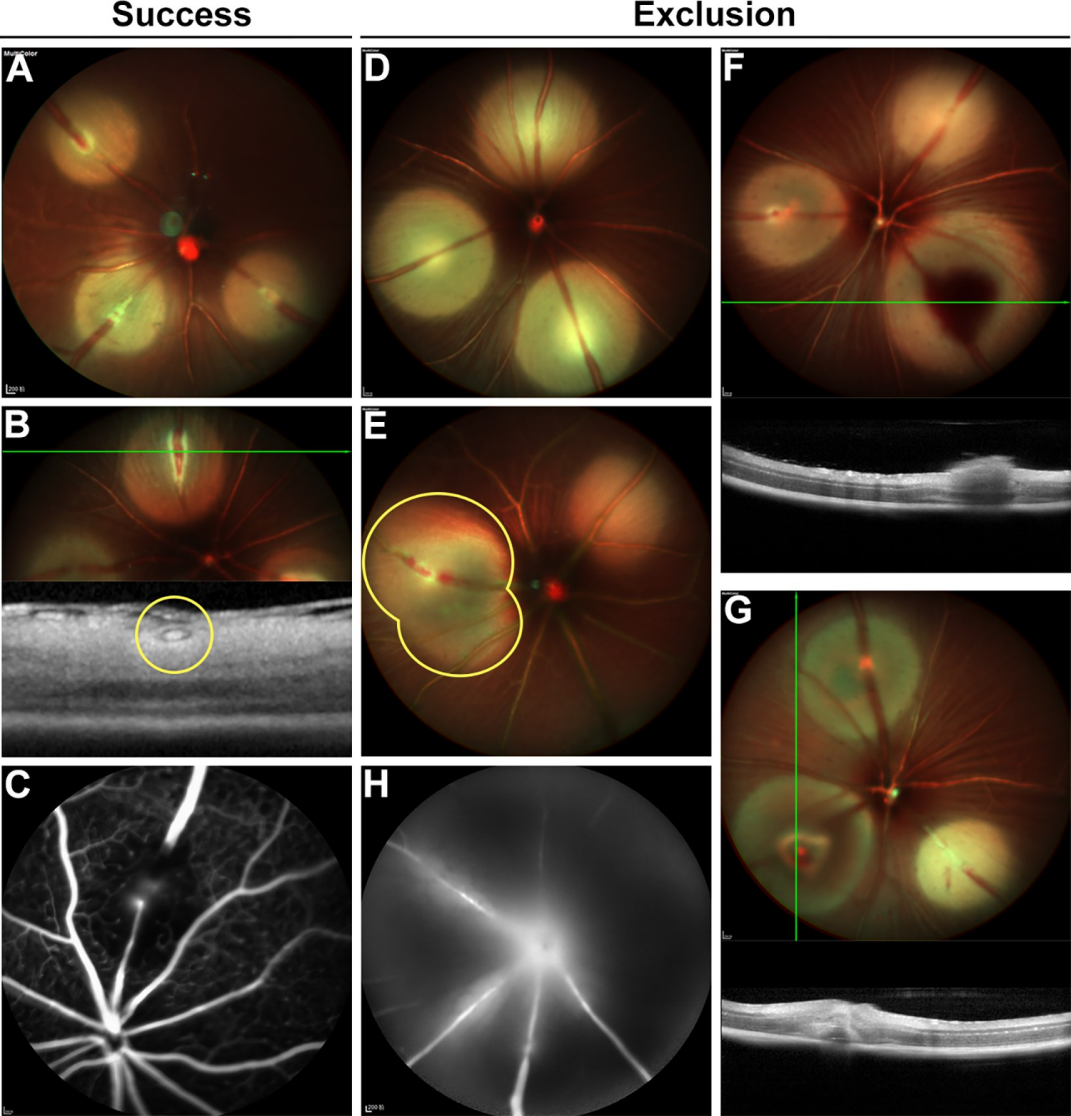
Success and exclusion criteria for the RVO mouse model. Success criteria for RVO after photocoagulation: (A) extreme vein stenosis on SLO, (B) photothrombosis on SLO and the corresponding hyperreflective lump within the venous lumen on OCT, (C) venous filling defect on FFA. Exclusion criteria during the laser procedure and follow-up phase: (D) unoccluded veins on SLO, (E) irregularity of the laser spot on SLO, (F) hemorrhage of the target vein on SLO and OCT, (G) outer retina damage as the formation of evaporative bubbles on SLO and OCT during the laser procedure; (H) artery involvement manifested as retinal arteriovenous occlusion on FFA in the follow-up phase. Abbreviations: RVO, retinal vein occlusion. SLO, scanning laser ophthalmoscopy. OCT, optical coherence tomography. FFA, fundus fluorescein angiography.

The successful construction of an RVO mouse model was assessed during the photocoagulation procedure and the follow-up period, based on the fulfillment of success criteria and avoidance of exclusion indicators, as shown in Fig 2. Definite major vein occlusion post-laser procedure was a prerequisite, confirmed by extreme vein stenosis to near invisibility on SLO (Fig 2A), or photothrombosis on SLO and the corresponding hyperreflective lump within the venous lumen on OCT (Fig 2B). FFA was applied for cases with ambiguous judgments based on the combination of Mcolor and IR, where RVO presented as a venous filling defect, sometimes accompanied by local fluorescein leakage (Fig 2C). Distinct exclusion criteria were applied to photocoagulation and follow-up procedures. For photocoagulation, exclusions included unoccluded veins (Fig 2D), damage to fundus structures including irregular laser spots (Fig 2E) caused by inadequate anesthesia and eye movement, target vein hemorrhage (Fig 2F) and formation of outer retina evaporative bubbles (Fig 2G) from misoperation in laser procedure. Follow-up exclusions involved excessive retinal ischemia with artery involvement, manifested as retinal arteriovenous occlusion (Fig 2H) as well as refractive media opacity unsuitable for imaging tests.

### Optimal laser power of 100mW for an acute RVO mouse model

**Fig 3:**
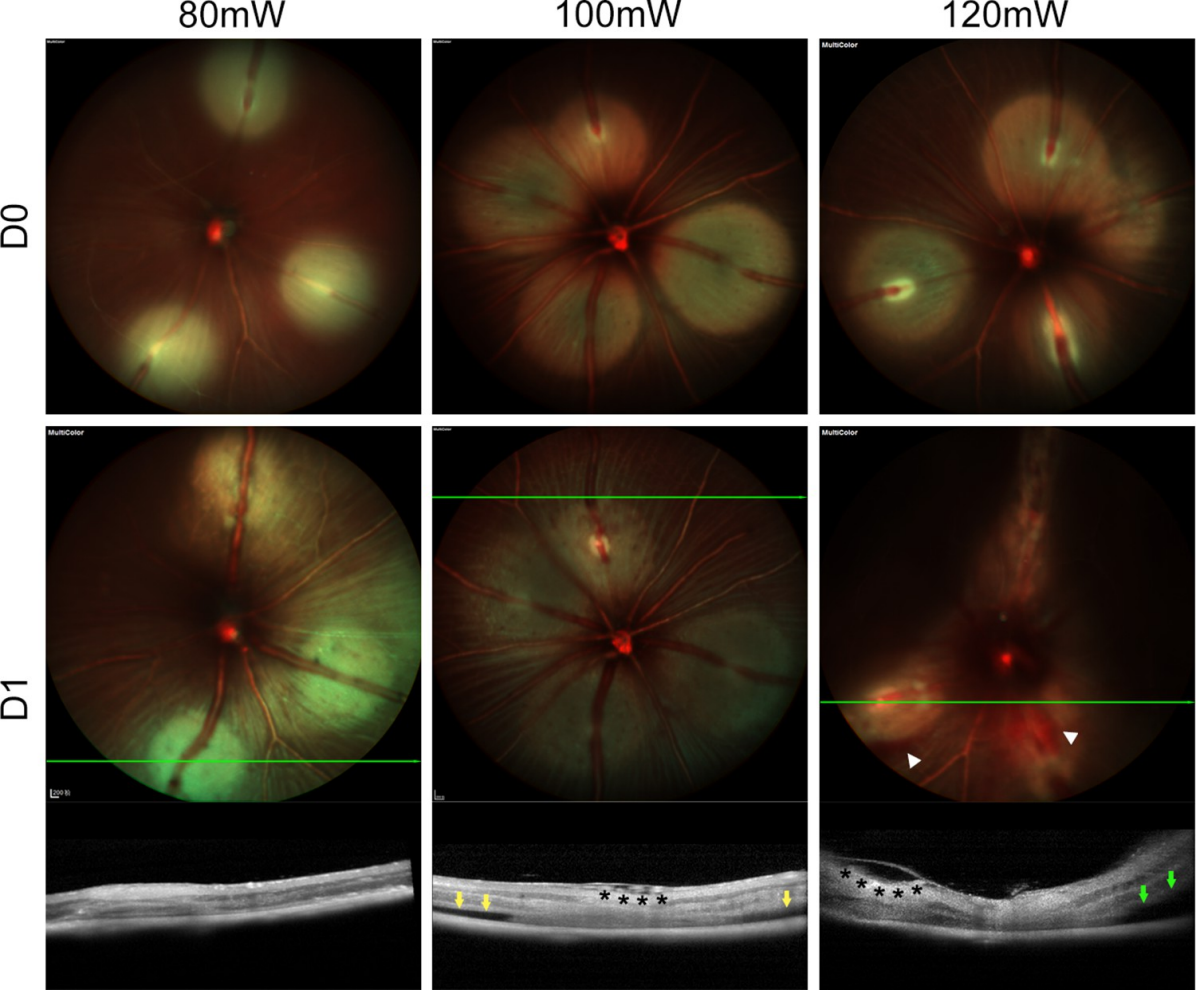
Typical fundus changes of 80mW, 100mW, and 120mW groups after RVO. Post-photocoagulation RVO was all achieved in 80mW, 100mW and 120mW groups by SLO. 1 day after RVO, 3 groups exhibited typical retinopathies on SLO and OCT. The 80mW group mainly developed venous recanalization on SLO. The 100mW group displayed cystoid edema (black asterisk) in NFL-GCL and superficial RD (yellow arrow) on SLO. The 120mW group exhibited intraretinal hemorrhage on SLO (white arrowhead), severe RD on both SLO (dark areas) and OCT (green arrow), as well as cystoid edema on OCT (black asterisk). Abbreviations: RVO, retinal vein occlusion. SLO, scanning laser ophthalmoscopy. OCT, optical coherence tomography. NFL-GCL, nerve fiber layer-ganglion cell layer. RD, retinal detachment.

Venous occlusion occurred after photocoagulation in the 80mW, 100mW, and 120mW groups with varying severity of retinopathies (Fig 3). The 80mW group mainly experienced partial vein recanalization, as indicated by the clear widening of the photocoagulated venous segment on Mcolor or IR, or the disappearance of the filling defect on FFA when judgments were challenging using the aforementioned two examination modes. Both the 100mW and 120mW groups presented retinal cystoid edema in nerve fiber layer-ganglion cell layer (NFL-GCL). Additionally, the 100mW group exhibited superficial RD as slight neuroretina-retinal pigment epithelium (RPE) separation on OCT. The 120mW group had retinal hemorrhage and severe RD, with the latter marked by dark areas on Mcolor and severe neuroretina-RPE separation on OCT.

Based on quantitative analysis of acute RVO lesions in the three groups (Table 1), a laser energy of 100mW was recommended for the acute RVO model due to its ability to achieve a balance among vein recanalization, exudative RD and characteristic retinopathies during the follow-up process. Venous recanalization and severe RD should be controlled. The former was a significant side reaction, while the latter was an overreaction compromising follow-up imaging quality and an exclusion criterion in retinal thickness quantification. Meanwhile, characteristic retinopathies of intraretinal hemorrhage and retinal cystoid edema required some incidence. The laser energy of 120mW had a primary advantage in maintaining venous occlusion over the other two groups, which could be applied to chronic RVO models.

**Table 1:** Comparison of retinopathy incidence among the 80mW, 100mW and 120mW groups after RVO.

	Venous recanalization		Exudative RD		Cystoid edema	Intraretinal hemorrhage
	D1	D7	Superficial and severe	Proportion of severe		
80mW	37.9% (11/29)^**α**^	73.9% (17/23)	13.8% (4/29)^**ρ**^	25.0% (1/4)	20.7% (6/29)^**δ**^	0% (0/29)^**γ**^
100mW	16.7% (4/24)^**αβ**^	68.2% (15/22)	62.5% (15/24)^**ν**^	53.3% (8/15)	58.3% (14/24)^**φ**^	41.7% (10/24)^**λ**^
120mW	0% (0/22)^**β**^	50.0% (11/22)	72.7% (16/22)^**ν**^	81.3% (13/16)	63.6% (14/22)^**φ**^	72.7% (16/22)^**λ**^

Chi-square test followed by Bonferroni multiple comparisons on venous recanalization, exudative RD, retinal cystoid edema, and intraretinal hemorrhage on day 1 (and day 7) were applied among the three laser energy groups. Three groups for retinopathy were denoted by superscript letters, indicating a non-identical incidence rate. Different superscript letters between any two groups signified a significant difference. Abbreviations: RVO, retinal vein occlusion. RD, retinal detachment.

### Acute RVO progression in SLO

**Fig 4:**
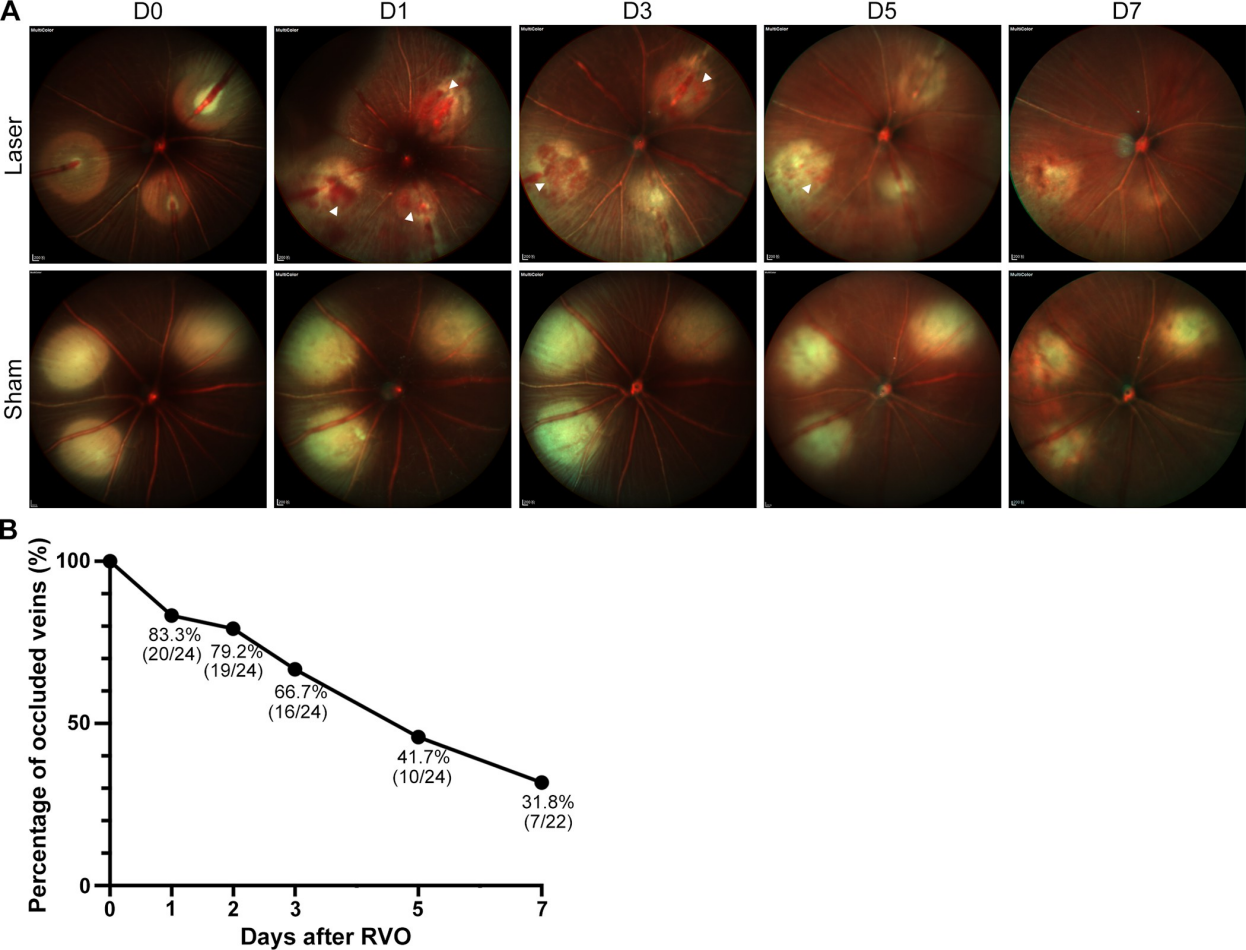
Retinopathy progression of acute RVO in SLO. (A) The progression of intraretinal hemorrhage in the 100mW acute RVO model (Laser group) compared with its control (Sham group) after photocoagulation (Day 0) and at days 1, 3, 5, and 7 after RVO. In the Laser group, intraretinal hemorrhage (white arrowhead) and exudative RD (dark area) both occurred on day 1 after RVO. The former gradually resolved during days 3 to 5 after RVO, while the latter resolved on day 3 after RVO. No intraretinal hemorrhage nor RD was observed in the Sham group. (B) The venous occlusion rate trend steadily decreased during the follow-up period in the acute RVO model. Abbreviations: RVO, retinal vein occlusion. SLO, scanning laser ophthalmoscopy. RD, retinal detachment.

Continuous SLO was performed on the 100mW acute RVO model (Laser group) and its control (Sham group) (Fig 4A). In the Laser group, intraretinal hemorrhage manifested as perivenous small patches, occurring consistently 1 day after RVO, with individual variations in complete absorption recovery from day 2 to 5. When exudative RD reached a severe degree, it might be visualized as non-opacified areas on Mcolor. Dilatation and tortuosity of target veins could persist throughout the follow-up period. The Sham group exhibited only edge blur and size reduction of laser spots progressing over time. The venous recanalization trend of the acute RVO model was analyzed in Fig 4B, revealing a gradual decrease from 83.3% on day 1 to 31.8% on day 7 in obstructed veins. No significant difference in intraretinal hemorrhage incidence was observed between individuals with venous obstruction and reperfusion on day 1 after RVO (p = 0.165), suggesting that persistent venous occlusion may be unnecessary for intraretinal hemorrhage.

### Acute RVO progression in OCT

**Fig 5:**
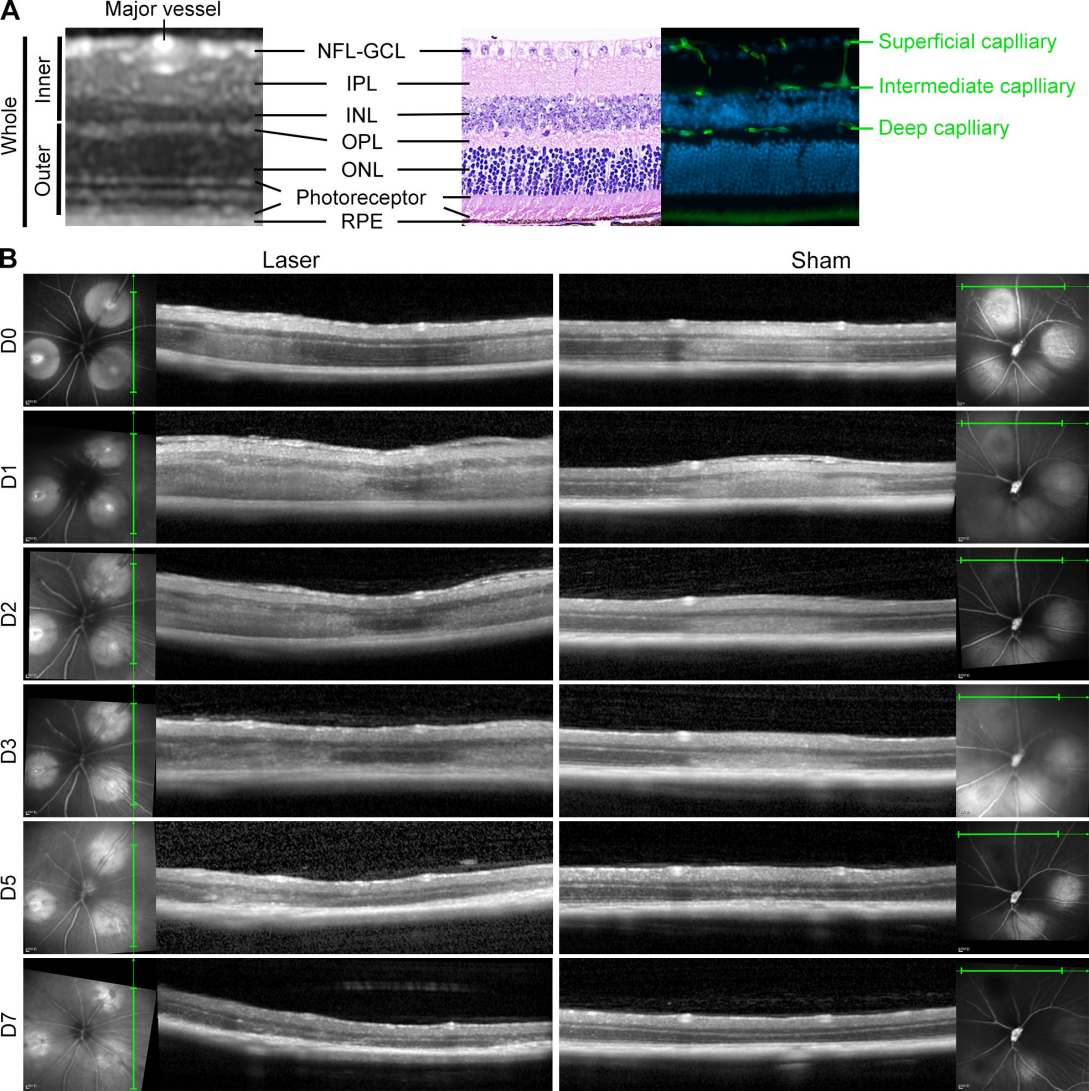
Retinopathy progression of acute RVO in OCT. (A) Comparative OCT, histology and immunofluorescence of retinal cross-section revealed retinal layers and vascular distribution. The whole retina included NFL-GCL, IPL, INL, OPL, ONL, photoreceptor layer and RPE. NFL-GCL to INL constituted the inner retina, while OPL to the photoreceptor layer constituted the outer retina. NFL-GCL contained both major vessels and superficial retinal capillaries, intermediate capillaries were located at the IPL-INL junction, and deep capillaries were situated at the INL-OPL junction. (B) Progression of retinal edema to atrophy of the 100mW acute RVO model (Laser group) compared with the control (Sham group) after photocoagulation (day 0) and at day 1, 2, 3, 5 and 7 after RVO. Abbreviations: RVO, retinal vein occlusion. OCT, optical coherence tomography. NFL-GCL, nerve fiber layer-ganglion cell layer. IPL, inner plexiform layer. INL, inner nuclear layer. OPL, outer plexiform layer. ONL, outer nuclear layer. RPE, retinal pigment epithelium.

Comparison of OCT, histology and immunofluorescence images revealed the structural features and vascular distribution of a normal retinal cross-section (Fig 5A). The whole retina consisted of NFL-GCL structure complex, inner plexiform layer (IPL), inner nuclear layer (INL), outer plexiform layer (OPL), outer nuclear layer (ONL), photoreceptor layer and RPE, while the inner retina ranged from NFL-GCL to INL, and the outer retina was composed of OPL, ONL and photoreceptor layer. Both major vessels and superficial retinal capillaries were located within NFL-GCL, intermediate capillaries were found at the IPL-INL junction, and deep capillaries lay at the INL-OPL junction.

A continuous OCT test was conducted on the Laser and the Sham groups (Fig 5B). Both groups progressed from retinal edema and thickening to atrophy and thinning during the follow-up period. However, the Laser group exhibited a stronger response, often accompanied by cystoid edema in NFL-GCL and/or INL and exudative RD, both occurring 1 day after RVO. The cystoid edema resolved rapidly, disappearing within 2 to 3 days after RVO. The recovery time of exudative RD depended on its severity, with superficial RD subsiding by day 2, while the subretinal fluid in severe RD persisted until day 3 to 5 after RVO. Disorganization of the outer retina was commonly observed in both groups. Venous recanalization seemed to have minimal impact on cystoid retinal edema or exudative RD, as there was no significant difference in the incidence of these pathologies at vein occlusion and recanalization sites (p = 1.000, p = 0.165).

### Retinal thickness trend of acute RVO

**Fig 6:**
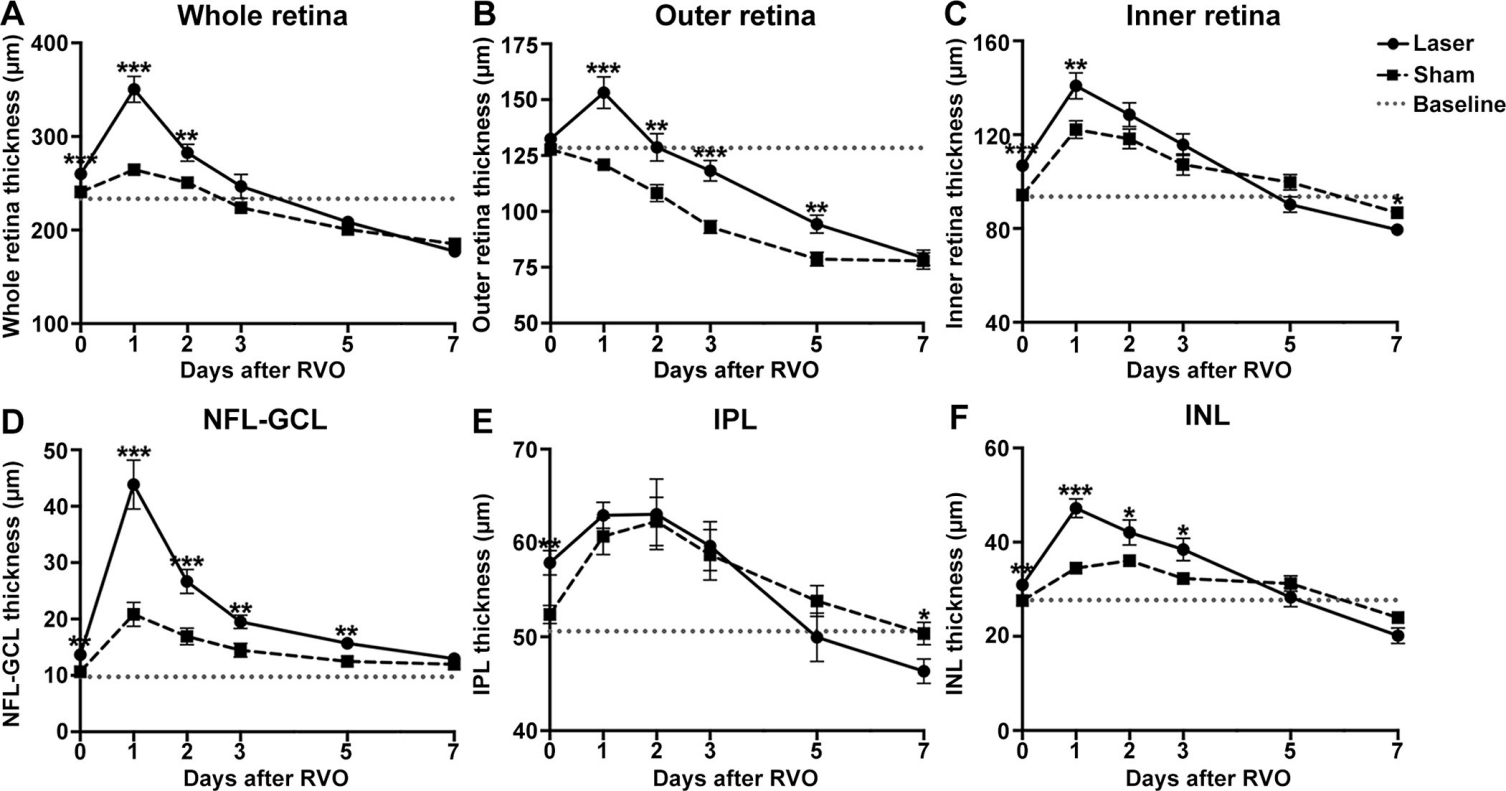
Retinal thickness trend of the acute RVO. Retinal layer thickness after photocoagulation (day 0) and at days 1, 2, 3, 5, and 7 after RVO was presented as mean±SEM. The 100mW acute RVO model (Laser group) was compared with the control (Sham group) at each time point using unpaired Student’s t-test or Mann-Whitney U test for datasets that did not adhere to the normal distribution: *p<0.05, **p<0.01, ***p<0.001. Normal retinal thickness was analyzed as the baseline. (A-F) Thickness trend of whole retina, outer retina, inner retina, NFL-GCL, IPL, and INL, respectively. A consistent peak in thickness on day 1 after RVO occurred across all retinal layers, with significant differences compared to the control group except for INL. Abbreviations: RVO, retinal vein occlusion. NFL-GCL, nerve fiber layer-ganglion cell layer. IPL, inner plexiform layer. INL, inner nuclear layer.

The thickness trends of various retinal layers in the Laser and the Sham groups were analyzed, with normal retinal thickness as the baseline (Fig 6). The Laser group exhibited a consistent retinal overall thickness and individual layer trend, peaking on day 1 after RVO, and there were significant differences compared with the control group except for IPL. In the whole retina, statistically significant differences between the two groups persisted until day 2 after RVO (Fig 6A). The outer retina of the Laser group was significantly thicker than that of the Sham group within 5 days after RVO, despite atrophy in both groups by 3 days after RVO (Fig 6B). In the inner retina, both groups exhibited reduced thickness from the first day after RVO, and atrophy manifested by day 7, notably more severe in the Laser group (Fig 6C). No atrophy occurred in NFL-GCL for either group, and there was a significant difference in thickening in the Laser group compared to the Sham group within 5 days after RVO (Fig 6D). The thickness of IPL in the Laser group decreased after day 1, and was finally significantly thinner than the Sham group at day 7 after RVO (Fig 6E). In INL, the thickness of the Laser group was significantly higher than that of the Sham group within 3 days after RVO (Fig 6F).

Thickening of retinal layers 1 day after RVO was illustrated in Table 2. The NFL-GCL and INL showed the most pronounced thickening with thickness growth rates of 220.1% and 52.6%, respectively. The influence of venous recanalization on retinal thickness was concentrated within the first two days after RVO. The thickness of the inner retina and INL on day 1 after RVO, and the whole and outer retina on day 2 after RVO, decreased at venous recanalization sites compared to occlusion sites (p = 0.022, 0.015; p = 0.005, 0.002). Thereafter venous recanalization had no significant effect on retinal thickness except for the alleviation of INL atrophy on day 5 after RVO (p = 0.017) (S2 Table).

**Table 2:** Thickening of retinal layers on day 1 after RVO.

	Whole retina	Outer retina	Inner retina	NFL-GCL	IPL	INL
**Thickness growth rate**	34.8%	15.6%	31.8%	220.1%	8.7%	52.6%

Abbreviations: RVO, retinal vein occlusion. NFL-GCL, nerve fiber layer-ganglion cell layer. IPL, inner plexiform layer. INL, inner nuclear layer.

### Positive response of acute retinal edema to anti-VEGF therapy

**Fig 7:**
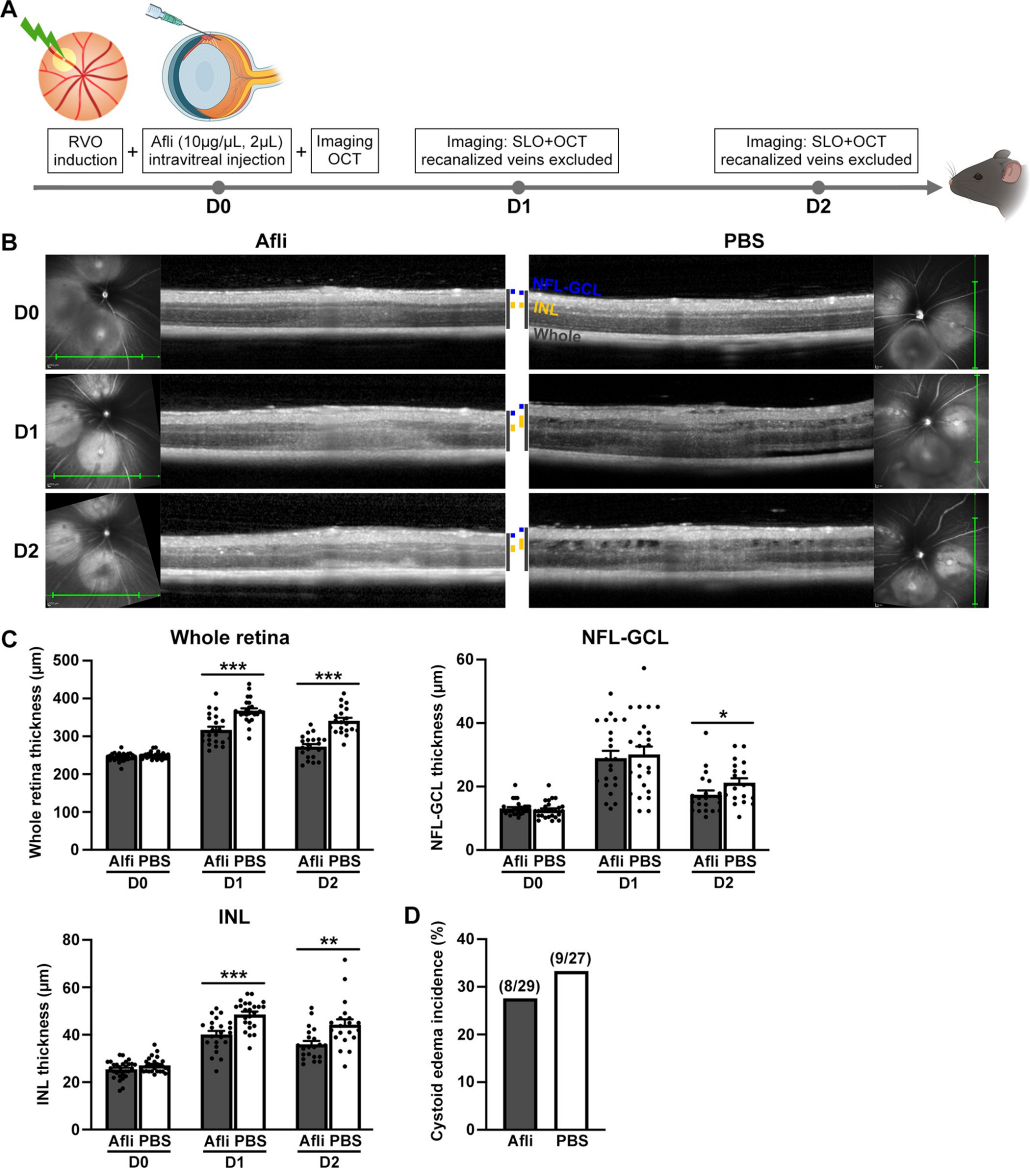
The efficacy of anti-VEGF treatment in alleviating acute retinal edema. (A) The schedule for anti-VEGF administration and efficacy evaluation in the acute RVO mouse model. On D0, after establishing the acute RVO model and conducting baseline OCT thickness measurements of the whole retina, NFL-GCL, and INL, immediate intravitreal injections of Aflibercept at 20 μg/μL, 2 μL per eye were administered. On D1 and D2, OCT scans were performed after SLO examinations to exclude recanalized veins, assessing the thickness of the aforementioned layers and the occurrence of cystic edema. (B) Representative images of Aflibercept alleviating acute retinal edema. Significant reductions in whole retinal and INL thickness were observed in the Aflibercept group at D1 and D2 compared to the PBS group. (C) Quantification of Aflibercept’s reduction of retinal thickness in the whole retina, NFL-GCL, and INL. At D1 and D2, the thickness of the whole retina and INL was significantly lower in the Aflibercept groups compared to the control group (P<0.05). (D) Aflibercept slightly reduced the incidence of cystoid retinal edema. The retinal layer thickness and the occurrence of retinal cystoid edema were presented as mean±SEM and incidence, respectively. The retinal layer thickness at each follow-up point in the Aflibercept group and PBS group was compared by unpaired Student’s t-test or Mann-Whitney U test for datasets that did not adhere to the normal distribution, while the incidence of cystoid retinal edema between the two groups was compared by Chi-square test: *p<0.05, **p<0.01, ***p<0.001. Abbreviations: RVO, retinal vein occlusion. Afli, Aflibercept. PBS, phosphate-buffered saline. OCT, optical coherence tomography. SLO, scanning laser ophthalmoscopy. NFL-GCL, nerve fiber layer-ganglion cell layer. INL, inner nuclear layer.

The protocol for assessing the responsiveness of acute retina edema to anti-VEGF intervention was developed based on the retinal thickness trend observed in the acute RVO model (Fig 7A). The study lasted two days and consisted of three time points: D0, D1, and D2. At D0, after inducing acute RVO, OCT baseline thickness evaluation of the whole retina, NFL-GCL, and INL was conducted, followed by the immediate injection of Aflibercept. On both D1 and D2, OCT evaluations of the aforementioned retinal layer thickness and cystic edema occurrence were administered following SLO examinations to exclude recanalized veins. Acute retinal edema responded positively to aflibercept, exhibiting decreased retinal thickness compared to the control group at both follow-up points, especially in the INL and the entire retina, with statistical significance (P<0.05) (Fig 7B and 7C). Additionally, a slight reduction in the incidence of cystic edema was observed in the Aflibercept group (Fig 7D).

### A chronic RVO mouse model featuring vasculature alterations

**Fig 8:**
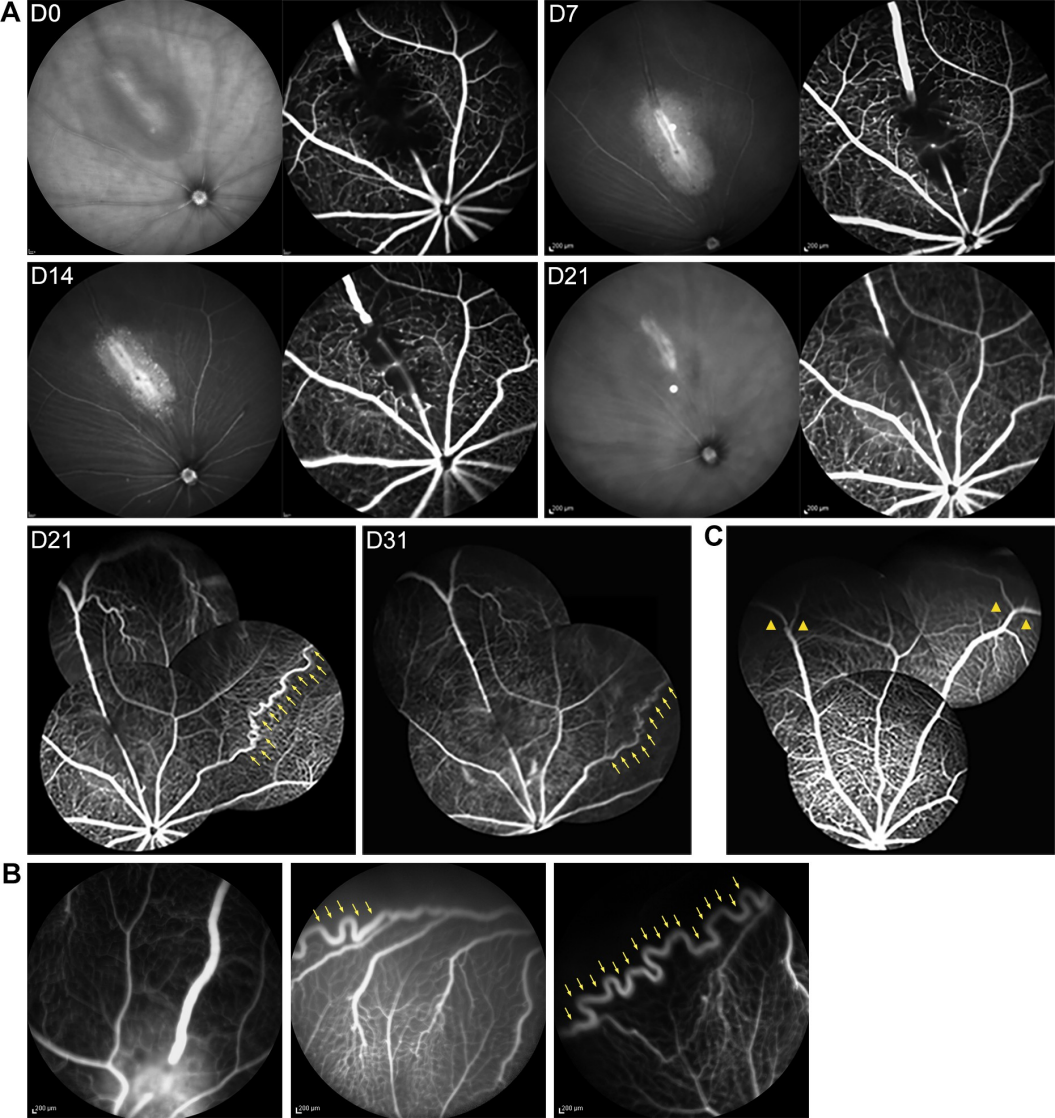
Retinal vasculature and perfusion in the chronic RVO. The chronic RVO model mainly manifested as changes in the morphology of the major vein and its branches. (A) IR and (or) FFA were performed after photocoagulation (day 0) and days 7, 14, 21 and 31 after RVO for retinal vasculature and perfusion evaluation. The length of the occluded venous segment decreased from days 7 to 21 after RVO, but rebounded by day 31 after RVO. The nonperfusion area of the laser spot reduced during the follow-up phase, with none observed in the peripheral retina. Tortuous change (yellow arrow) in an adjacent major vein was observed on day 21 and persisted until day 31 after RVO, without affecting the distal end of the occluded vein. (B) The tortuous change was also found in the peripheral branches of the occluded veins. (C) Normal retinal vasculature and perfusion as the control. The major retinal vein emitted two branches (yellow arrowhead) in the peripheral retina, with some connecting to adjacent ones, accompanied by retinal capillaries on FFA. Abbreviations: RVO, retinal vein occlusion. IR, Infrared mode. FFA, fundus fluorescein angiography.

A chronic RVO mouse model characterized by persistent venous occlusion was established for the evaluation of retinal vasculature and perfusion alterations (Fig 8). The photocoagulation energy, number of shots and range per vein were increased to extend the duration of venous occlusion, with the exclusion of recanalized veins at each time point. FFA in the normal retina revealed each major peripheral vein emitting two branches, some connecting to adjacent ones and forming an equatorial venous ring, alongside distributed retinal capillaries (Fig 8C). After photocoagulation (Day 0), a long segmental vein obstruction and surrounding retinal laser damage appeared as a narrow line circled a light-colored laser spot on IR, which coincided with interrupted contrast agent filling and capillary nonperfusion at the corresponding site on FFA. Distal vein dilatation was observed on both tests. The length of the interrupted fluorescein-filling vein gradually shortened, and the surrounding nonperfusion area reduced on FFA from day 7 to 21 after RVO, matching the decrease of laser spot area on IR. However, on day 31 after RVO, the venous occlusion segment lengthened compared to day 21. The distal end of the occluded vein remained dilated until day 14 and mostly normalized to the proximal end level by day 21 after RVO. The tortuous change was observed in the adjacent major vein at day 21, without affecting the distal end of the occluded vein, and persisted until day 31 after RVO (Fig 8A). Moreover, the same tortuous change was also observed in the peripheral branches of the occluded veins (Fig 8B). Neither retinal arterial occlusion (RAO) nor other abnormal fundus responses were observed during follow-up.

## Discussion

Establishing an RVO mouse model involves achieving complete occlusion of major veins and mild inner retinal edema within the laser spot, without causing damage to other retinal layers or fundus structure. Laser focus placement is crucial for successful photocoagulation, as indicated by clear reflection of the target venous wall at the specified point with a slight blurring of the fundus structure between large vessels. A forward-shifted laser focus raises the probability of vein narrowing rather than complete occlusion or photothrombosis. Moreover, intense irritation at the retina-vitreous interface may result in irreversible refractive media opacity, impacting subsequent imaging observations. A backward-shifted laser focus poses risks of damaging major veins and outer retinal structures. The former presents as venous hemorrhage, while the latter results in outer retinal vaporization bubbles or even rupture of Bruch’s membrane manifesting as fluorescein leakage on FFA similar to choroidal neovascularization [19]. Notably, *C57Bl/6J* mice are more susceptible to outer retina damage due to the inclination of energy absorption by pigments in RPE.

The systematic circulation time of photosensitizers, although not specified in the majority of studies (S1 Table), serves as a necessary parameter and a major source of variability among individuals, affecting the successful construction and retinopathy progression of RVO models. In Miyagi et al.’s [20] study, a 20-minute interval between Rose Bengal application and RVO laser induction prevented the excessive ischemia dominated by RAO that occurred without an interval, and facilitated the development of characteristic flame-like retinal hemorrhage. The photodynamic effects on tissue primarily depend on the local concentration of the photosensitizer during its temporal distribution process [21]. After intravenous administration of the Rose Bengal, FFA at 22 seconds reveals higher fluorescence in retinal arteries than veins [21], highlighting the heightened susceptibility to RAO without an interval. Additionally, Colón Ortiz et al. determined that 10min is the optimal choice for the consistency of venous occlusion by comparing the modeling effects of 5, 10, 15 and 20min [5]. Nonetheless, some studies applied the parameter of 3 min to achieve venous occlusion [10, 12, 22, 23]. Based on the findings of various researchers, we concluded that a circulation time of 10 to 20 minutes is optimal and set a parameter of 15 min. A certain amount of practice should be undertaken to ensure the coordination of steps involving tail vein photosensitizer injection, assessment of anesthesia depth, and accurate positioning of the laser focus for consistent photosensitizer circulation time.

Clinical subtypes of RVO also include central RVO (CRVO), branch RVO (BRVO), and hemi-RVO, distinguished by the location and number of occluded veins [24]. The existing RVO mouse model mainly aims to simulate BRVO and CRVO. BRVO is induced by photocoagulation of one to several major veins, while CRVO is induced by photocoagulation at the optic disc or all major veins [7, 12]. However, due to the venous occlusion rate of 40–50% after photocoagulation [5], combined with the influence of venous recanalization, CRVO frequently presents as BRVO. Moreover, the large number of CRVO laser shots increases the risk of energy dispersion, leading to excessive retinal swelling and potential involvement of arterial occlusion [5, 7]. Hence, our model targets BRVO and imposes strict limits on the number of photocoagulated veins.

The application of multimodal imaging for fundus morphological and perfusion assessment enables ongoing observation of RVO progression and prevents false-positive results from RD and structural damage in histologic tests [13]. However, it requires high-quality refractive media. The combination of corneal moisturizers, rewarming devices, and anesthetic reversal agents during the imaging tests and anesthesia recovery alleviates stress on refractive media caused by post-anesthetic blinking reduction and hypothermia, repeated general anesthesia and pupil dilation in the follow-up period [25]. Furthermore, image quality can be optimized through proper planning of the inspection item sequence. Various laser wavelengths are utilized in multimodal imaging: OCT at 870 nm [26, 27], Mcolor (IR: 815 nm, green: 518 nm, blue: 486 nm), IR at 815 nm, and FA at 488nm. Their tissue penetration reduces as the laser wavelength decreases, increasing the demand for refractive media transparency. A recommended sequence involves conducting FFA first (when retinal perfusion assessment is required), followed by SLO, and concluding with OCT.

The acute RVO model progressed rapidly, with retinal morphological changes peaking at day 1 after RVO. Retinal edema was the hallmark pathological alteration, universally present and exhibiting a consistent progression pattern. The NFL-GCL and INL were primarily involved and thickened the whole retina with significant swelling and cyst formation, pronounced within 2 days after modeling, followed by gradual subsidence and atrophy within a week. However, the acute course diverges from the chronic and recurrent features of macular edema [24], indicating potential differences in their pathological mechanisms. Moreover, the time constraints imposed by the short duration of acute edema on the intervention window could influence therapeutic efficacy. These factors may collectively limit the applicability of the acute RVO model. Anti-VEGF agents, including bevacizumab, ranibizumab, aflibercept, brolucizumab, and faricimab, constitute the first-line therapy for macular edema and operate by stabilizing the vasculature to reduce leakage [28]. We believe that confirming the responsiveness of acute edema to anti-VEGF treatment can enhance our understanding of its pathological mechanisms and elevate the model’s utility as a positive control for evaluating the efficacy of both established and emerging treatments for RVO. Several studies have indicated a significant upregulation of VEGF expression in the RVO mouse model during the early follow-up period [12, 13, 29–31], and Ichiyama et al. [32] have verified that Aflibercept effectively neutralizes mouse VEGF, providing a prerequisite for the efficacy of anti-VEGF agents in acute edema. In our acute RVO model, retinal edema showed a favorable response to Aflibercept treatment, with significant reductions in INL and the whole retinal thickness on days 1 and 2 after RVO compared to controls. Similarly, Miyagi et al. observed relief of INL edema with anti-VEGF treatment on Day 3 [20]. These positive findings confirmed the acute RVO model’s strong clinical relevance, effectively recapitulating the pathological progression of vasogenic edema attributed to increased leakage of the inner blood-retinal barrier (iBRB) in macular edema [33], allowing for further application in the studies of RVO pathogenesis and efficacy assessment.

While the pathological mechanism underlying the acute RVO model remains incompletely understood, it inevitably involves elevated hydrostatic pressure within the obstructed vein [33], contributing to various characteristic pathological changes. This pressure is transmitted to iBRB, comprising major vessels and superficial capillaries in the NFL-CGL, and intermediate and deep capillaries adjacent to the INL. Increased fluid passes the iBRB and accumulates in the NFL-GCL and INL, leading to cyst formation, which broadens the previous understanding that cystoid retinal edema is limited to albino mice species [13]. In cases of excessive pressure, capillary rupture occurs with intraretinal hemorrhage formation. Hypoxia is also involved in the pathological process of acute RVO. The hypoxia probe can produce positive results as early as 2 hours and lasts 8 days after RVO [9, 11]. Disorganization of retinal inner layers on OCT has been previously reported [6, 8], and we observed outer retinal layer disorganization, indicating potential multilevel and deep capillary ischemia, respectively [34, 35]. However, given the prevalence of controls in our study, more consideration was given to direct damage from the laser.

One challenge with the acute RVO model is the high incidence of exudative RD and the prevalence of severe grading, exceeding typical clinical observations. In our acute model, exudative RD was categorized as superficial or severe depending on severity. Superficial RD exhibited mild neuroretinal-RPE separation visible only on OCT, considered an acceptable pathological change. Its absence in controls and presence in the clinical course [36] elucidated the pathology’s mechanism of increased iBRB leakage as the primary factor, rather than direct laser damage to the retina. Severe RD demonstrated pronounced neuroretinal-RPE separation on OCT and even dark areas on SLO, suggesting an overreaction with the involvement of other mechanisms. Photodynamic injury to the posterior segment structures during RVO induction might be the leading cause of exacerbated RD, with its effects largely dependent on the local concentration of photosensitizer [21, 37]. Photosensitizer is distributed temporally in fundus tissue during systemic circulation. After the intravenous injection of Rose Bengal, accumulation occurs in the choroid and RPE within 5 minutes, with increased content observed in the outer retina at 20 minutes, and detection in the inner retina after 60 minutes [21]. Based on our 15-minute parameter, photodynamic injury primarily affected the choroid and the RPE. Choroidal involvement, characterized by both choriocapillary occlusion and non-occlusion injury, is deemed a necessity for the development of exudative RD [38]. Additionally, RPE injury can occur independently with choriocapillary ischemia or in combination with photodynamic free radical injury [39]. Either choriocapillary occlusion alone or RPE injury combined with non-occlusion choriocapillary injury can lead to the formation of serous RD [38]. Unfortunately, exudative RD was prevalent across studies [5, 6, 13, 15], with the highest rate exceeding 90% [13], indicating that the existing photosensitizer systematic circulation duration parameters could not prevent its occurrence. Furthermore, laser energy may influence the development of exudative RD. The comparison of our 80mW, 100mW, and 120mW groups suggested that the incidence of exudative RD increased with higher laser energy, accompanied by a higher percentage of severe RD. Therefore, we hypothesized that moderately prolonging the photosensitizer systematic circulation time and reducing laser energy could attenuate the formation of exudative RD, mitigating interference from photodynamic tissue injury on the pathological progression of RVO and reducing the exclusion visits due to OCT follow-up criteria.

Venous recanalization is a pivotal deficiency in the acute RVO mouse model. Although no influence was observed on the incidence of intraretinal hemorrhage, cystoid retinal edema or exudative RD, recanalization within 2 days after RVO significantly alleviated thickening in various retinal layers. Therefore, maintaining early-phase venous occlusion in the acute RVO mouse model is crucial. Our model showed a steady decline in venous obstruction rates throughout the follow-up period, improving the extensive venous recanalization on day 2 after RVO in the previous study [9]. The venous state in the hyperacute phase after photocoagulation determines venous obstruction. The occlusion state undergoes rapid changes within the initial 10 minutes following the laser procedure, and maintaining this state during this period ensures a minimum duration of 24 hours [5]. In clinical practice, arteriosclerosis significantly contributes to the pathological process of RVO, characterized by an arteriovenous crossover sign resulting from arterial thickening, venous compression, degeneration, and hypercoagulability at the arteriovenous crossing [40, 41]. The absence of arterial system abnormalities may explain venous recanalization in the short term in purely photochemical RVO animal models. However, our chronic RVO mouse model significantly prolonged the duration of venous obstruction and reduced the rate of venous recanalization by increasing the energy, number of shots, and range of photocoagulation to extend venous occlusion scope from ‘point’ to ‘segment’. A few veins were recanalized within 1 week after RVO, but the vein status tended to stabilize and no recanalization happened 2 weeks after RVO. Maintaining sustained venous occlusion ensured stable retinal perfusion levels and prevented the interference of ischemia-reperfusion injury with the progression of chronic RVO.

Chronic RVO is characterized by alterations in retinal vasculature and perfusion. Major veins may exhibit the formation of vascular sheaths and collateral vessels [40]. Microangiopathy represents the principal pathological change and serves as an indicator of retinal perfusion, as evidenced by nonperfusion, capillary dilation, microaneurysms, collateral vessels, and neovascularization on FFA [42]. Nonperfusion, especially in the peripheral area where higher pressure is needed to fill capillaries, is necessary for neovascularization and indicates the onset of ischemic retinopathy [43]. Our chronic RVO model achieved nonischemic retinopathy by persistent obstruction of a single major vein. The effects of increased venous hydrostatic pressure on the retinal major veins and microvasculature were not parallel. Tortuous dilation of the distal portion of the occluded vein progressed to the equatorial branches and adjacent major veins. However, no peripheral retinal nonperfusion occurred during the follow-up period, with the nonperfusion area at the laser spot continuing to shrink. Our findings aligned with Fuma et al.’s [13] study, suggesting that the retinal nonperfusion area primarily arose from direct laser damage to the retinal microvasculature and recovered during the chronic course, although there is some enlargement observed at day 30 compared to day 7 after RVO in their model. Takahashi et al. [10] applied OCT angiography and found that collateral vessels in the deep retina layer gradually open 3 days after RVO, connecting the distal end of the obstructing vein to and nearest adjacent vein, or bypassing the obstruction point to connect to the same vein, which may represent as a compensatory mechanism to relieve retinal ischemia.

We speculate that the number of occluded veins is an important factor affecting retinal perfusion. Ischemic CRVO has a larger nonperfused area than BRVO, exceeding 5 and 10 disc areas, respectively [44]. Similarly, our acute model with obstruction of 3 veins had occasional excessive ischemia with retinal artery involvement, whereas the chronic model with 1 vein blocked did not exhibit ischemic retinopathy or RAO complications. Retinal capillary perfusion relies on the pressure gradient between the retinal artery/arteriole and vein/venule [43]. Abundant occlusion of retinal veins elevates hydrostatic pressure in the capillary bed, leading to the cessation of blood flow in retinal arteries when it exceeds arterial perfusion pressure [45]. Moreover, RVO-induced retinal edema further compresses arteries and exacerbates RAO [45]. In our chronic RVO model, an additional burden on capillary perfusion came from the increased non-perfusion area of the laser spot, induced by the elevated laser energy and expanded photocoagulation range to a single vein. To mitigate its effect, we restricted the number of photocoagulated veins to one and regulated the number of laser shots to be roughly equivalent to the total in the acute model. However, efforts to induce ischemic retinopathy by increasing the number of occluded veins may face challenges due to the difficulty in avoiding the confounding factor of excessive photocoagulation.

The characteristic changes in retinal vasculature and perfusion in the chronic RVO model determine its potential applications. In the preclinical efficacy evaluation of retinal vascular diseases, morphological improvement represents a crucial indicator. Current research primarily focuses on microvascular alterations, with limited attention to major vessels. However, Choi et al. [46] quantified and evaluated the dilatation and tortuosity of major retinal veins, clarifying the effectiveness of intravitreal dexamethasone implant in non-ischemic CRVO without macular edema for the first time. Therefore, we hypothesize that parameters of tortuosity and dilatation of the major veins in the chronic RVO model could serve as efficacy evaluation indicators, exerting a great impact on future research. Furthermore, with the increasing prevalence of electrophysiological techniques in preclinical research, assessments of preclinical efficacy will gradually demand visual functional recovery evaluations. Our chronic RVO model, characterized by non-ischemic retinopathy, could complement preclinical RVO studies dominated by ischemic retinopathy using the oxygen-induced retinopathy model [47]. Non-ischemic and ischemic RVO exhibit considerable differences in visual function and treatment response [48]. The need for comparing baseline levels and treatment outcomes in both retinal morphology and function between the two subtypes provides broad application prospects for the chronic RVO model. Additionally, non-ischemic RVO is conducive to investigating retinal compensation mechanisms in chronic ischemic-hypoxic conditions. Takahashi et al. observed gradual collateral vessel formation post-venous occlusion, followed by rapid regression upon vein reperfusion [10]. Therefore, it is speculated persistent venous obstruction in our chronic RVO model may stabilize collateral vasculature, facilitating comprehensive follow-up of its natural progression. Moreover, the stable obstruction of retinal veins sets the stage for investigating the long-term mechanisms of retinal response to ischemia-hypoxia at the cellular and molecular levels. With increasing research focus on ischemic-hypoxic brain injury, attention has shifted to the retina as an extension of the central nervous system. Cascade activation of multiple signaling pathways induced by excitotoxicity, oxidative stress, inflammation, and mitochondrial dysfunction is implicated in chronic cerebral under-perfusion, leading to immune cell recruitment and activation, and necrosis/apoptosis of vascular endothelial and supportive cells, as well as neurons [49, 50]. Some of these mechanisms have been validated in the retina, offering novel therapeutic targets for RVO [6, 17, 51, 52].

## Conclusion

We confirmed that laser energy influenced retinopathies in RVO, and adopted different model parameters for acute and chronic RVO with a slit lamp system. The acute RVO model targeted an appropriate degree of morphological changes, with a peak response observed 1 day after the laser procedure. The most representative pathologies included retinal swelling and thickening, accompanied by dense retinal cyst formation, intraretinal hemorrhage, and the development of exudative RD, with the most severe involvement occurring in NFL-GCL, where the iBRB was present, and in INL near its distribution. Extensive venous recanalization was avoided within the first 2 days after modeling, which typically led to a reduction in retinal thickness. Retinal edema demonstrated a positive response to classical anti-VEGF therapy, affirming the clinical significance of this model for therapeutic assessment and investigations into pathological mechanisms. Chronic RVO was characterized by a stable obstruction of a single major vein and exhibited nonischemic retinopathy with predominant macrovascular involvement and negligible microangiopathy. Marked venous tortuosity and dilation can be quantified and utilized alongside electrophysiological functional assessment as an indicator for therapeutic evaluation. A stable venous occlusion state allows for the study of reactive and compensatory mechanisms under conditions of chronic retinal hypoperfusion. Parameters for acute and chronic RVO models may require adjustments based on specific laboratory conditions. When introducing new observational indicators, it is advisable to set up a control group to exclude the potential influence of the laser itself.

## Supporting information

S1 Table Parameters of the RVO mouse model. (DOCX)

S2 Table Comparison of retinal layer thickness for venous occlusion and recanalization. (DOCX)
