# Supplementary material for: Exploring laser-induced acute and chronic retinal vein occlusion mouse models: Development, temporal in vivo imaging, and application perspectives
Source: PLoS One. 2024 Jun 17;19(6):e0305741. doi: 10.1371/journal.pone.0305741 (PMC11182531; doi:10.1371/journal.pone.0305741)
Supplement: S1 Table — (DOCX) [file pone.0305741.s001.docx]

**S1 Table. Parameters of the RVO mouse model**

| **Research group** | **RVO subtype** | **Animal species** | **Photosensitizer** | | | **Laser photocoagulation parameters** | | | | | | |
| --- | --- | --- | --- | --- | --- | --- | --- | --- | --- | --- | --- | --- |
|  |  |  | **Type** | **Dose** | **Circulation time (min)** | **Wavelength**  **(nm)** | **Power**  **(mW)** | **Duration**  **(s)** | **Spot size (µm)** | **Burns per vein** | **Number of vein(s)** | **Distance from optic disk** |
| Hideaki Hara (2017)[1] | BRVO | ddY;  C57BL/6J  (2021)[2] | RB | 8mg/ml;  8mg/ml, 0.15ml  (2021)[2] | UK;  20 (2021)[2] | 532 | 50;  130  (2021)[2] | 5;  0.3 (2021)[2] | 50 | 10~15;  20~30  (2021)[2] | 3 | 3PD |
| Carol M. Tory (2020)[3] | CRVO | C57BL/6J | RB | 5mg/ml, 0.15ml;  37.5mg/kg (2021)[4] | 15; 10 (2021)[4] | 532 | 100 | 1 | 50 | 3 | 3~6; 2~3 (2021)[4] | 375µm |
| Kanji Takahashi  (2021)[5] | BRVO | BALB/cA | RB | 5mg/ml, 0.15ml | 3 | 532 | 200 | 0.1 | 100 | UK | 1 | 1~2PD |
| Martin S. Zinkernagel  (2015)[6] | BRVO, CRVO; BRVO (2017[7]; 2020[8]) | BALB/c AnNCrl | RB | 5mg/ml, 0.15ml | 3 | 532 | 160 | 0.8~2.5; 0.8 (2017[7]; 2020[8]) | 50 | 2~5 | 1 or all | 1~2PD; 2PD (2020)[8] |
| Takayuki Shindo  (2019)[9] | CRVO | C57BL/6J | RB | 40mg/kg | UK | 532 | 50 | 3 | 50 | Several times | UK | At the optic disk |
| Florian Sennlaub  (2015)[10] | BRVO | C57BL/6J | Fluo | 1%, 100µl | UK | 532 | 200 | 0.5 | 50 | 7~12 | 1 | 2~3PD |
| Gottfried Martin  (2018)[11] | CRVO | C57BL/6J | EY | 200mg/kg | UK | 532 | 50 | 2.5 | 50 | 3~6 | all | UK |
| Michael J. Allingham  (2018)[12] | BRVO | C57BL/6J | RB | 66mg/kg | None | 532 | 80 | UK | 50 | 3～7 | 1 | 1PD |
| Md. Imam Uddin  (2017)[13] | BRVO | C57BL/6J | RB | 40mg/kg | UK | UK | 50 | 1 | 50 | 2～3 | 1～2 | Close to optic disk |
| Koh-Hei Sonoda  (2007)[14] | BRVO | C57BL/6J | RB | 40mg/kg | UK | 530.9 | 50 | 3 | 50 | 2～3 | 3～5 | Close to optic disk |
| Martin Friedlander  (2005)[15] | CRVO | BLAB/cByJ | UK | UK | UK | UK | 180 | 1 | 50 | UK | 1 | Close to optic disk |
| Eric Vicaut (2003)[16] | BRVO | C57 BLKS | None | None | None | UK | 0.1 | 1 | 50 | 2～6 | 1 | 2～3PD |

Abbreviations: RVO: retinal vein occlusion; CRVO: central retinal vein occlusion; BRVO: branch retinal vein occlusion; PD, papillary diameter; RB, Rose Bengal; Fluo, fluorescein; EY, Eosin Y; UK: unknown.

**References**

1. Fuma S, Nishinaka A, Inoue Y, Tsuruma K, Shimazawa M, Kondo M, et al. A pharmacological approach in newly established retinal vein occlusion model. Scientific reports. 2017;7:43509.

2. Miyagi S, Nishinaka A, Yamamoto T, Otsu W, Nakamura S, Shimazawa M, et al. Establishment of a pigmented murine model abundant with characteristics of retinal vein occlusion. Exp Eye Res. 2021;204:108441. Epub 2021/01/17. doi: 10.1016/j.exer.2021.108441. PubMed PMID: 33453278.

3. Avrutsky MI, Ortiz CC, Johnson KV, Potenski AM, Chen CW, Lawson JM, et al. Endothelial activation of caspase-9 promotes neurovascular injury in retinal vein occlusion. Nature Communications. 2020;11(1):3173.

4. Colón Ortiz C, Potenski A, Lawson JM, Smart J, Troy CM. Optimization of the Retinal Vein Occlusion Mouse Model to Limit Variability. Journal of Visualized Experiments: JoVE. 2021;(174).

5. Takahashi H, Nakagawa K, Yamada H, Mori H, Oba S, Toyama K, et al. Time course of collateral vessel formation after retinal vein occlusion visualized by OCTA and elucidation of factors in their formation. Heliyon. 2021;7(1):e05902. Epub 2021/01/22. doi: 10.1016/j.heliyon.2021.e05902. PubMed PMID: 33474512; PubMed Central PMCID: PMCPMC7803649.

6. Ebneter A, Agca C, Dysli C, Zinkernagel MS. Investigation of retinal morphology alterations using spectral domain optical coherence tomography in a mouse model of retinal branch and central retinal vein occlusion. PloS one. 2015;10(3):e0119046.

7. Ebneter A, Kokona D, Schneider N, Zinkernagel MS. Microglia Activation and Recruitment of Circulating Macrophages During Ischemic Experimental Branch Retinal Vein Occlusion. Investigative ophthalmology & visual science. 2017;58(2):944-53.

8. Jovanovic J, Liu X, Kokona D, Zinkernagel MS, Ebneter A. Inhibition of inflammatory cells delays retinal degeneration in experimental retinal vein occlusion in mice. Glia. 2020;68(3):574-88.

9. Hirabayashi K, Tanaka M, Imai A, Toriyama Y, Iesato Y, Sakurai T, et al. Development of a Novel Model of Central Retinal Vascular Occlusion and the Therapeutic Potential of the Adrenomedullin-Receptor Activity-Modifying Protein 2 System. The American Journal of Pathology. 2019;189(2):449-66.

10. Dominguez E, Raoul W, Calippe B, Sahel J-A, Guillonneau X, Paques M, et al. Experimental Branch Retinal Vein Occlusion Induces Upstream Pericyte Loss and Vascular Destabilization. PloS one. 2015;10(7):e0132644.

11. Martin G, Conrad D, Cakir B, Schlunck G, Agostini HT. Gene expression profiling in a mouse model of retinal vein occlusion induced by laser treatment reveals a predominant inflammatory and tissue damage response. PloS one. 2018;13(3):e0191338.

12. Allingham MJ, Tserentsoodol N, Saloupis P, Mettu PS, Cousins SW. Aldosterone Exposure Causes Increased Retinal Edema and Severe Retinopathy Following Laser-Induced Retinal Vein Occlusion in Mice. Investigative ophthalmology & visual science. 2018;59(8):3355-65.

13. Uddin MI, Jayagopal A, McCollum GW, Yang R, Penn JS. In Vivo Imaging of Retinal Hypoxia Using HYPOX-4-Dependent Fluorescence in a Mouse Model of Laser-Induced Retinal Vein Occlusion (RVO). Investigative ophthalmology & visual science. 2017;58(9):3818-24.

14. Zhang H, Sonoda K-H, Qiao H, Oshima T, Hisatomi T, Ishibashi T. Development of a new mouse model of branch retinal vein occlusion and retinal neovascularization. Japanese Journal of Ophthalmology. 2007;51(4):251-7.

15. Ritter MR, Aguilar E, Banin E, Scheppke L, Uusitalo-Jarvinen H, Friedlander M. Three-Dimensional In Vivo Imaging of the Mouse Intraocular Vasculature during Development and Disease. Investigative Opthalmology & Visual Science. 2005;46(9):3021 %U <http://iovs.arvojournals.org/article.aspx?doi=10.1167/iovs.05-0153>.

16. Paques M, Tadayoni R, Sercombe R, Laurent P, Genevois O, Gaudric A, et al. Structural and Hemodynamic Analysis of the Mouse Retinal Microcirculation. Investigative Opthalmology & Visual Science. 2003;44(11):4960 %U <http://iovs.arvojournals.org/article.aspx?doi=10.1167/iovs.02-0738>.
