# Supplementary material for: Exploring laser-induced acute and chronic retinal vein occlusion mouse models: Development, temporal in vivo imaging, and application perspectives
Source: PLoS One. 2024 Jun 17;19(6):e0305741. doi: 10.1371/journal.pone.0305741 (PMC11182531; doi:10.1371/journal.pone.0305741)
Supplement: S2 Table — (DOCX) [file pone.0305741.s002.docx]

**S2 Table. Comparison of retinal layer thickness for venous occlusion and recanalization.**

|  |  | **Whole retina** | **Outer retina** | **Inner retina** | **NFL-GCL** | **IPL** | **INL** |
| --- | --- | --- | --- | --- | --- | --- | --- |
| **D1** | **Venous occlusion** (n=12) | 360.84±14.05 | 155.33±7.70 | 146.00±6.78 | 43.76±4.40 | 63.53±1.64 | 49.94±2.05 |
|  | **Venous recanalization** (n=4) | 319.05±34.25 | 146.60±18.23 | 125.60±4.02 | 44.15±12.80 | 61.20±2.85 | 39.08±2.65 |
|  | **Comparison between groups** | P=0.198 | P=0.611 | P=0.022 | P=0.978 | P=0.486 | P=0.015 |
| **D2** | **Venous occlusion** (n=12) | 295.93±8.81 | 136.80±6.38 | 133.18±5.86 | 28.68±2.49 | 65.27±4.90 | 42.83±3.35 |
|  | **Venous recanalization** (n=4) | 242.85±6.85 | 104.25±5.33 | 114.60±7.53 | 20.73±2.74 | 56.43±0.72 | 39.68±4.21 |
|  | **Comparison between groups** | IP=0.005 | P=0.002 | P=0.118 | P=0.109 | P=0.101 | P=0.627 |
| **D3** | **Venous occlusion** (n=14) | 243.92±18.53 | 119.79±5.27 | 119.46±6.42 | 18.94±1.01 | 61.83±3.66 | 39.26±3.16 |
|  | **Venous recanalization** (n=7) | 252.14±12.26 | 115.11±9.70 | 108.31±5.26 | 20.61±2.99 | 55.34±2.09 | 36.79±3.64 |
|  | **Comparison between groups** | P=0.771 | P=0.648 | P=0.274 | P=0.612 | P=0.247 | P=0.637 |
| **D5** | **Venous occlusion** (n=11) | 212.37±4.21 | 99.87±4.96 | 86.93±3.77 | 15.56±0.78 | 50.64±2.72 | 23.44±2.40 |
|  | **Venous recanalization** (n=12) | 205.03±7.22 | 89.23±5.87 | 93.33±5.48 | 15.88±1.02 | 49.34±4.36 | 32.77±2.63 |
|  | **Comparison between groups** | P=0.391 | P=0.185 | P=0.355 | P=0.808 | P=0.807 | P=0.017 |
| **D7** | **Venous occlusion** (n=6) | 172.80±5.34 | 80.48±5.95 | 76.57±4.17 | 13.47±1.35 | 47.35±2.33 | 16.70±2.83 |
|  | **Venous recanalization** (n=13) | 179.11±5.53 | 78.64±4.41 | 80.91±3.01 | 12.76±0.73 | 45.86±1.62 | 21.71±1.96 |
|  | **Comparison between groups** | P=0.425 | P=0.812 | P=0.421 | P=0.621 | P=0.609 | P=0.167 |

Abbreviations: NFL-GCL, nerve fiber layer-ganglion cell layer. IPL, inner plexiform layer. INL, inner nuclear layer.
